# The Application of Nanotechnology for the Diagnosis and Treatment of Brain Diseases and Disorders

**DOI:** 10.3389/fbioe.2021.629832

**Published:** 2021-03-02

**Authors:** Ebenezeri Erasto Ngowi, Yi-Zhen Wang, Lei Qian, Yasmeen Ahmed Saleheldin Hassan Helmy, Bright Anyomi, Tao Li, Meng Zheng, En-She Jiang, Shao-Feng Duan, Jian-She Wei, Dong-Dong Wu, Xin-Ying Ji

**Affiliations:** ^1^Henan International Joint Laboratory for Nuclear Protein Regulation, School of Basic Medical Sciences, Henan University, Kaifeng, China; ^2^Kaifeng Municipal Key Laboratory of Cell Signal Transduction, Henan Provincial Engineering Centre for Tumor Molecular Medicine, Henan University, Kaifeng, China; ^3^Department of Biological Sciences, Faculty of Science, Dar es Salaam University College of Education, Dar es Salaam, Tanzania; ^4^Brain Research Laboratory, School of Life Sciences, Henan University, Kaifeng, China; ^5^International Joint Center for Biomedical Innovation, School of Life Sciences, Henan University, Kaifeng, China; ^6^School of Nursing and Health, Institutes of Nursing and Health, Henan University, Kaifeng, China; ^7^School of Pharmacy, Institute for Innovative Drug Design and Evaluation, Henan University, Kaifeng, China; ^8^School of Stomatology, Henan University, Kaifeng, China; ^9^Kaifeng Key Laboratory of Infection and Biological Safety, School of Basic Medical Sciences, Henan University, Kaifeng, China

**Keywords:** nanotechnology, brain diseases and disorders, diagnosis, treatment, nanoparticles

## Abstract

Brain is by far the most complex organ in the body. It is involved in the regulation of cognitive, behavioral, and emotional activities. The organ is also a target for many diseases and disorders ranging from injuries to cancers and neurodegenerative diseases. Brain diseases are the main causes of disability and one of the leading causes of deaths. Several drugs that have shown potential in improving brain structure and functioning in animal models face many challenges including the delivery, specificity, and toxicity. For many years, researchers have been facing challenge of developing drugs that can cross the physical (blood–brain barrier), electrical, and chemical barriers of the brain and target the desired region with few adverse events. In recent years, nanotechnology emerged as an important technique for modifying and manipulating different objects at the molecular level to obtain desired features. The technique has proven to be useful in diagnosis as well as treatments of brain diseases and disorders by facilitating the delivery of drugs and improving their efficacy. As the subject is still hot, and new research findings are emerging, it is clear that nanotechnology could upgrade health care systems by providing easy and highly efficient diagnostic and treatment methods. In this review, we will focus on the application of nanotechnology in the diagnosis and treatment of brain diseases and disorders by illuminating the potential of nanoparticles.

## Introduction

Brain diseases and disorders refer to a large group of health conditions affecting the brain including injuries, infections, tumors, and neurological disorders. Based on the 2015 statistics, brain diseases and disorders are the main cause of disabilities and the second leading cause of mortality with more than 250.7 million disability-adjusted life-years and 9.4 million deaths (GBD 2015 Neurological Disorders Collaborator Group, 2017). By definition, the term “brain diseases” encompasses a group of medical conditions that are usually transmittable and commonly caused by external forces such as viruses, bacteria, and so on (Ghosh and Higgins, 2018), whereas “brain disorders” include non-transmittable but commonly inheritable medical conditions caused by the disruption of the normal body structure and functioning as a result of birth defects or genetic malfunctions (Borsche et al., 2020). Brain diseases include viruses/bacteria/fungi/parasite-caused brain infections (BIs), whereas disorders include conditions such as multiple sclerosis (MS), autism spectrum disorder (ASD), and Alzheimer disease (AD). Despite their differences, the two terms are regularly used interchangeably. The most notable features of brain diseases and disorders include deterioration of cognitive, motor, and behavioral functions resulting from the impairment of neurological activities. The treatment of these conditions has been hindered by the complexity and sensitivity of the organ. Some of the diseases including bacterial and fungal BI can be cured by specific antibiotics if discovered in initial states, or vaccines can be applied to prevent their onset (Baccarini et al., 2020); however, others such as neurodegenerative disorders have no exact cures. The physical, chemical, and electric barriers prevent the entrance of materials including most drugs into the brain (Janzer and Raff, 1987; Butt and Jones, 1992; Boulton et al., 2002). Previously, potential drugs used to be dissolved in the solvents that could disrupt the blood–brain barrier (BBB) such as ethanol, polysorbate 80 (PS-80), and dimethyl sulfoxide in order to increase their penetration and sensitivity (Hanig et al., 1972; Azmin et al., 1985; Butt and Jones, 1992). In recent years, nanoparticles (NPs)–based treatments have emerged as the potential therapy for brain diseases and disorders due to easy transportability across the BBB, a credit of their unique features such as small size, selectivity, less toxicity, biodegradability, and solubility (Broadwell et al., 1982; Rabanel et al., 2020).

**FIGURE 1:**
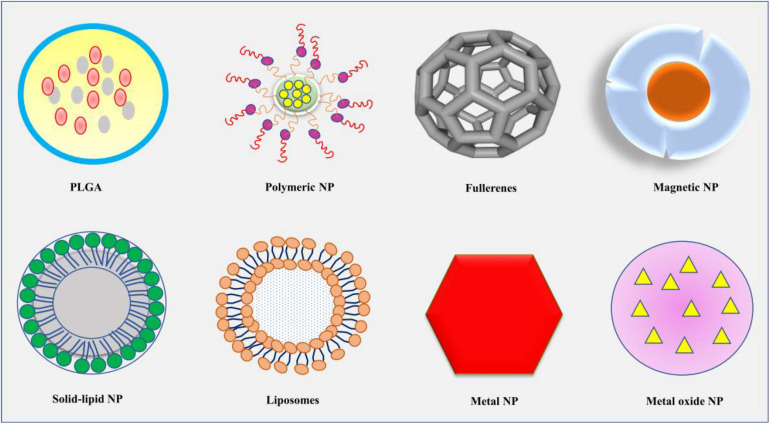
The diagrammatic presentation of some of the common shapes of NPs, including PLGA, polymeric NP, fullerenes, magnetic NP, solid-lipid NP, liposomes, metal NP, and metal oxide NP. NP, nanoparticle; PLGA, polylactide-co-glycoside.

**FIGURE 2:**
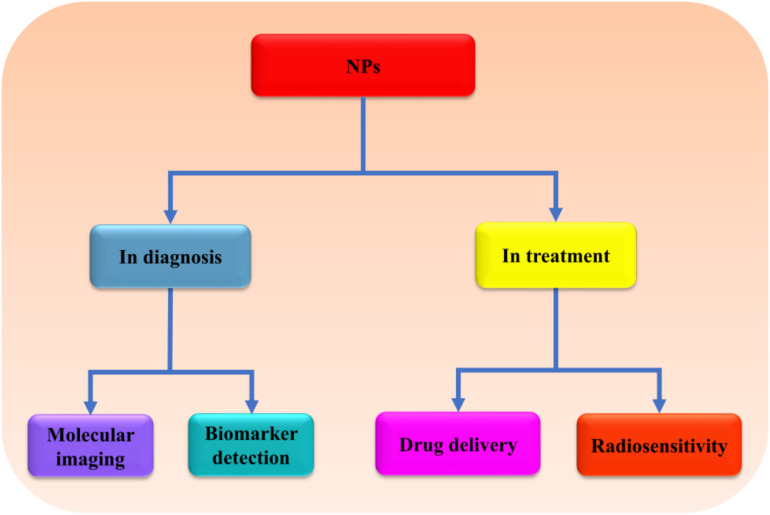
The possible application of NPs in brain therapy. NPs can be used in diagnosis as well as treatment of brain diseases and disorders due to their high sensitivity, specificity, and ability to cross BBB. NPs, nanoparticles; BBB, blood–brain barrier.

NPs refer to smallest particles usually within the size range of 1–100, at most less than 1,000 nm (Narayanan and Sakthivel, 2011). The particles are formed from natural or artificial manipulation of compounds or metals. So far, different kinds of NPs have been produced such as metal and metal oxides, liposomal, polymeric, fullerenes, nanoemulsions, solid–lipid (SL), polylactide-co-glycoside (PLGA) NPs, and so on, with varying physical and chemical properties (Figure 1; Narayanan and Sakthivel, 2011; Djordjevic et al., 2015; Ding et al., 2016; Lee et al., 2016; Dong et al., 2018; Rasouli and Tabrizian, 2019; Matsuno et al., 2020). The synthesized NPs have been applied in different fields such as cosmetics, agriculture, and medicines (Lu et al., 2015; Karny et al., 2018; Hydbring and Du, 2019; Katebi et al., 2019). In medicines, NPs are used in the diagnosis and treatments of different diseases especially the ones that are deeply seated such as metastatic cancers, brain tumor, and neurodegenerative disorders (Figure 2; Zhang et al., 2016; Li et al., 2019a). Because of the challenges facing the effective and efficient delivery of drugs in such conditions, NPs appear to be an important discovery that may enhance the effectiveness and efficiency of potential drugs. In response to the current rise in number of NPs that have shown enormous potential in the treatment of brain diseases and disorders, this review will summarize the application of NPs in the treatment of brain diseases and disorders, as well as the challenges facing this novel discovery. Hereby, the therapeutic potential of several NPs including metal, lipid, polymeric, coffee, SL, chitosan (CS), magnetic, rare-earth (RE), fullerenes, poly(butyl cyanoacrylate) (PBCA), PLGA, betulinic, and liposomal NPs will be discussed.

## The Role of BBB and Its Influence on Therapy Feasibility

BBB is a physical barrier formed by endothelial cells (ECs) with the main role of maintaining and regulating the movement of nutrients and other essential materials to the brain, thereby protecting its integrity. The ECs are located on the outer and inner sides of the closely packaged tight junctions that touch the outer EC membranes and prevent easy penetration of materials (Reese and Karnovsky, 1967). Some of the main functions of the BBB include the regulation of the flow of materials in and out of the brain, ionic balance, and protection from the diffusion of circulating agents, neurotransmitters, xenobiotics, and other substances that can affect the integrity of the brain (Abbott et al., 2010). Studies show that poor permeability of molecules across the BBB is significantly associated with high electrical and chemical [P-glycoproteins (P-gp)] resistance (Crone and Christensen, 1981; Butt et al., 1990; Li et al., 2018). Some of the vital regulators of tight junctions’ activities identified are cyclic adenosine monophosphate and astrocytes (Rubin et al., 1991; Hurst and Clark, 1998). In brain diseases and disorders, the BBB is highly disrupted, resulting into unregulated diffusion of molecules, leading to further brain damage (Dallasta et al., 1999; Algotsson and Winblad, 2007). Because the BBB prevents the entrance of materials basing on their size and solubility, most of the potential drugs fail to penetrate because they do not meet the required criteria (Pardridge, 2012). One of the common techniques used to improve the transportation of drugs across the barrier is the temporal disruption using focused ultrasound (McDannold et al., 2012), although the mechanism involved and the effect of the technique on an already disrupted barrier are yet to be elucidated. Otherwise, the search for a non-disruptive technique for transportation of drugs to the brain has also been given a high priority, and in recent times, NPs have proven to be efficient in fulfilling the role.

## Advantages of NPs for Brain Therapy

### NPs Have Small Particle Size That Facilitates Their Penetration Across the BBB

**FIGURE 3:**
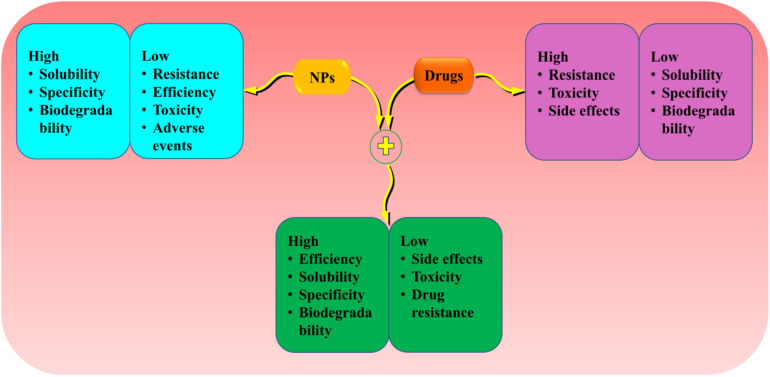
The advantage of loading drugs into the NPs compared to individual treatments. Encapsulation of potential drugs offers advantages into the NPs and drugs features to provide high effectiveness and efficiency. NPs, nanoparticles.

Crossing the BBB and blood–cerebrospinal fluid (CSF) barrier has been the main challenges hindering the treatment of brain diseases and disorders. The efflux of materials across BBB is carefully mediated by P-gp; hence, its downregulation is implicated with the progression of neurodegenerative disorders and tumor (Henson et al., 1992; van Assema et al., 2012; Jeynes and Provias, 2013). The inhibition of P-gp improves the penetration of drugs across the BBB and their subsequent effects (Jablonski et al., 2014). NPs of PBCA have been reported to suppress P-gp-mediated phenytoin resistance in rats (Fang et al., 2016). Moreover, a recent study shows that encapsulation of andrographolide (a neuroprotective drug) into SL NPs increases its permeability to the BBB compared to free drug (Graverini et al., 2018). In summary, the above data indicate that NPs can enhance the penetration of potential drugs and increase their target ability by regulating p-gp (Figure 3).

### NPs Have Low Toxicity and Can Be Used to Improve the Toxicity of Conventional Drugs in Their Targeted Cells

The therapeutic efficacy of most drugs is affected by their cytotoxicity. The brain toxicity induced by NPs is much less compared to conventional therapy. For example, in cerebral ischemia/reperfusion model, intranasal administration of PLGA NPs is reported to be highly effective in transporting a mitoNEET ligand inhibitor NL-1 with no toxicity (Saralkar et al., 2020). Further studies have also shown that the encapsulation of cytotoxic drug such as amphotericin B (antifungal drug), thioridazine (antipsychotic drug), and sorafenib (anticancer drug) into NPs markedly improves their toxicity index by enhancing drug solubility, bioavailability, and sustained release (Tang et al., 2015; Vibe et al., 2016; Li et al., 2020).

Alternatively, NPs can also increase the cytotoxicity of conventional drugs in their targeted area, e.g., tumor cells. In a recent study, treatment with polyethylene glycol (PEG)-modified silica (Si) NPs has been shown to increase the cytotoxicity of anticancer drug, 3N-cyclopropylmethyl-7-phenyl-pyrrolo-quinolinone as compared to free drug in an *in vitro* model (Morillas-Becerril et al., 2020). Despite the observed potential of NPs, some of these compounds can also result in cytotoxicity, including Si NPs whose effect is shown to be influenced by the porosity and size of the particles (Mohammadpour et al., 2019). Apart from the use of particle sizes and shapes that show less toxicity, another method that can be used to improve the efficiency of NPs is the addition of PEG also known as PEGylation (Mendonça et al., 2016; Abakumov et al., 2018). In summary, the evidences above indicate significant reduction in drug toxicity when loaded into some NPs as compared to when administered freely; although in some cases the encapsulation can result in increased cytotoxicity, the event can be reduced with PEGylation or alteration of particle size and porosity.

### NPs Improve the Solubility and Bioavailability of Conventional Drugs

Other parameters that are essential in determining the efficacy of a drug is solubility and bioavailability. Solubility is the ability of the drug to dissolve, whereas bioavailability is the extent to which the drug can reach the systemic blood circulation and subsequently the targeted site (Chow, 2014; Alany, 2017). Unlike solubility, the factors affecting drug bioavailability can be drug-related or body-related. Some of them include age, sex, gut pH, genetics, drug dosage, and formulation. Because of the importance of these parameters, improving both of them can lead to better drug efficacy and ultimately treatment of the disease. It has been reported that silver (Ag) NPs can significantly enhance the solubility of methane and ethane in water, with the solubility shown to increase with NPs mass loading (Rahmati-Abkenar and Manteghian, 2020). A recent study indicates that the loading of hydrophobic drug, carvedilol, into CS-sodium tripolyphosphate (STPP) NPs increases its bioavailability and promotes slow and sustained release of the drug (Sharma et al., 2019). Similarly, the oral bioavailability and solubility of curcumin (a polyphenol and turmeric compound) can be improved by loading into PEGylated SL NPs (Ban et al., 2020). The bioavailability and solubility of many other potential drugs such as astilbin, sorafenib, apigenin, and astaxanthin also have been reported to be improved following the encapsulation into NPs (Huang et al., 2019; Liu et al., 2019; Park et al., 2019; Zheng and Zhang, 2019). Together, the data above suggest that NPs can potentially increase the solubility and bioavailability of less-soluble drugs, therefore improving their efficacy.

### NPs Improve the Specificity and Biocompatibility of Conventional Drugs

The specificity and biocompatibility of the drug ensure effective delivery to the targeted site. Incorporating drugs into NPs help to substantially enhance these parameters. A recent evidence shows that chimeric antigen receptor T-cell membrane–encapsulated NPs have high specificity in targeting tumor cells by recognizing glycan-3 proteins, which are highly expressed in hepatocellular carcinoma cells with good biocompatibility and safety in normal cells (Ma et al., 2020). Biomimetic gold (Au) NPs stabilized by seaweed extracts have also been reported to be lethal in breast cancer cells MDA-MB-231 at the dose of less than 45 μg/mL while showing no effects on human embryonic kidney cells at 150 μg/mL, which confirmed the high biocompatibility and selectivity of the NPs (Jeyarani et al., 2020). In addition, specific antibody-loaded iron oxide (IO) NPs have shown high sensitivity and specificity, greater than 95 and 90%, respectively, in capturing amyloid β (Aβ) and Tau proteins in the serum and CSF-mimicking samples and about 80–90% in human whole blood samples as compared to the common antibody-conjugated magnetic micron beads, which show approximately only 20% specificity and sensitivity, suggesting the potential of the technique as a biomarker for dementias (Li et al., 2019b). Overall, the above data imply that NPs are highly specific and biocompatible and therefore can be used to deliver drugs to the targeted sites more efficiently.

## Nanotechnology in Brain Diseases and Disorders

### Molecular Imaging (MI)

MI is an important field in biomedical science associated with the analysis of pathogenesis or body functioning at the molecular level. The imaging techniques provide easy visualization, characterization, and quantification of activity of interest in the body with high sensitivity and specificity (Weissleder and Mahmood, 2001). It involves the use of advanced techniques of different capabilities including microscopy, bioluminescence imaging, ultrasound, X-ray radiography, magnetic resonance imaging, positron emission tomography, and single-photon emission computed tomography. MI techniques have proven to be useful in analysis and characterization of different brain diseases ranging from infections to brain tumors and neurological disorders (Mankoff, 2007; Aldossary et al., 2019; Bocan et al., 2019). The specificity of MI is enhanced by the use of contrast beacons known as probes (Schocke et al., 2002). Probes that bind to specific targets are called targetable probes, whereas the ones that react with specific indicators on their targets to produce a visible signal are termed as activatable probes. A previous study suggests that oligopeptides NPs can act as activatable probes because of their ability to produce fluorescence as a result of the stimulation by low pH of tumor microenvironment (Massoud and Gambhir, 2003). Besides, it has been shown that PS-80–coated PBCA dextran polymeric NPs can be used to transport targetable probes across the BBB, thereby facilitating the visualization of Aβ plaques in AD model (Zhao et al., 2014). A recent study also reports that sulfated dextran-coated IO NPs can effectively improve bioimaging of the activated microglia-induced brain inflammation by binding to the highly expressed class A scavenger receptors (Tang et al., 2018). In addition, it has also been shown that RE-doped NPs can be used in fluorescence imaging to facilitate the emission of short-wave infrared light after binding to integrin α Vβ3 (Naczynski et al., 2018). In summary, the information above indicates that NPs can be used to improve MI by delivering bioimaging probes or acting as probes themselves, confirming their importance in diagnosis of deep-seated tumor and brain diseases.

### Biomarker Detection

Biomarker is simply a detectable substance/indicator that is directly associated with a certain condition or state. The effectiveness of the biomarker to differentiate between healthy and unhealthy individuals and its specificity in characterization of the disease stage are key in management of diseases. Different biomarkers have been identified in brain diseases and disorders; however, their application is hindered by the lack of suitable techniques. NPs have shown to be useful in detecting key biomarkers of brain diseases and disorders with great efficiency. In traumatic brain injury (TBI) patients, plasma levels of ubiquitin-C-terminal hydrolase-L1 (UCH-L1) have been identified to be significantly elevated as compared to healthy individuals and therefore could serve as a potential biomarker for the condition (Posti et al., 2016). A recent study demonstrates that a novel method involving surface plasmon resonance of Au NPs can rapidly and effectively detect UCH-L1 biomarker in TBI patients with 100% sensitivity and specificity (Singh et al., 2018). Besides, Aβ levels have been markedly correlated with dementia and associated diseases (van Steenoven et al., 2019). Studies have shown that modified magnetic NPs can effectively and safely detect Aβ plaques in the mouse model of AD (Cheng et al., 2015; Zeng et al., 2018). Anticholesterol antibody-bound magnetic NPs have further been shown to be effective in detecting elevated cholesterol levels, which is also a key marker for AD (Fernández-Cabada and Ramos-Gómez, 2019). Moreover, a recent study indicates fluorescent NPs can be used to detect AD biomarkers including Aβ, inflammatory cytokines, and Tau proteins (Sun et al., 2021). Collectively, these data suggest that NPs are quick and effective in detecting biomarkers for brain diseases and disorders.

### Delivery of Drugs

Delivery of the potential drugs in the brain is one of the main challenges facing the treatment of brain diseases and disorders. The drug is supposed to be able to cross the BBB and reach the designated target without causing serious short- or long-term damage into the brain. The size and number of hydrogen bonds are among the factors preventing the transportation of the drugs across the BBB (Pardridge and Mietus, 1979; Pardridge, 2012). In recent years, NPs have gained a lot of attention due to their ability to cross the BBB and serve as a carrier for potential drugs. NPs show enhanced BBB penetrating capabilities and can be loaded with potential brain-targeting drugs (Lin et al., 2016; He et al., 2019; Sadegh Malvajerd et al., 2019). In addition, Au NPs have also been shown to be effective in delivering antibody across the BBB by binding to transferrin receptors, although the effect depends on the affinity and valency of the conjugated antibody (Johnsen et al., 2018). Intranasal delivery of huperzine A with lactoferrin-conjugated N-trimethylated CS-modified PLGA increases its bioavailability and retention time in the mouse model of AD (Meng et al., 2018). Together, these data imply that NPs can effectively and efficiently deliver different drugs across the BBB.

### Radiosensitization

**FIGURE 4:**
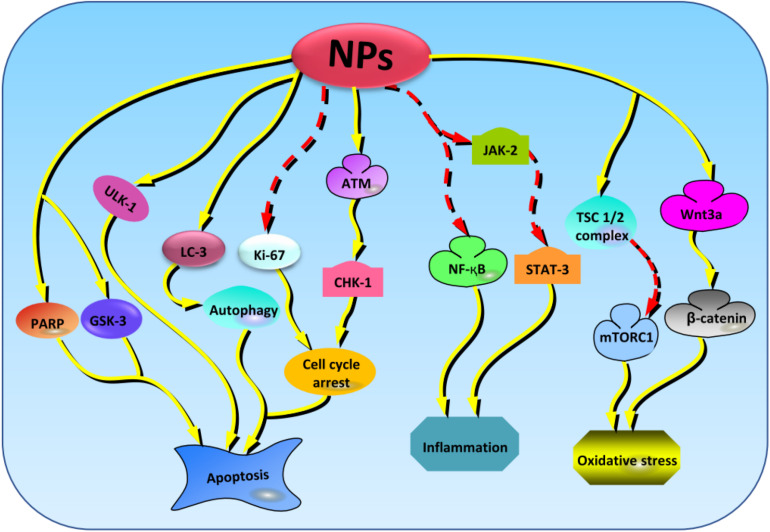
The key cellular markers targeted by NPs in regulating the oxidative stress, inflammation, and apoptosis activities. From the left to right; NPs stabilize the levels of PARP, GSK-3, and ULK-1 to regulate apoptosis. Next, NPs amplifies the expression of LC-3 to induce protective autophagy and regulate apoptosis. NPs also suppress the expressions of Ki-67 and promotes the activation of ATM/CHK-1 cascades resulting in promotion of apoptosis. NPs can also enhance the inhibition of NF-κB and JAK-2/STAT-3 signaling pathways to regulate inflammation. Besides, NPs elevate the levels of TSC1/2 complex and Wnt3a, thereby promoting the inhibition and activation of mTORC1 and β-catenin, respectively, and resulting in the stimulation of antioxidant activities. NPs, nanoparticles; PARP, poly(ADP-ribose) polymerase; GSK-3, glycogen synthase kinase-3; ULK-1, unc-51 like autophagy activating kinase-1; ATM/CHK-1, ataxia telangiectasia mutated/checkpoint kinase-1; NF-κB, nuclear factor κB; JAK-2/STAT-3, janus kinase 2/signal transducer and activator of transcription 3; TSC1/2, tuberous sclerosis protein complex 1/2; mTORC1, mammalian target of rapamycin complex 1.

The resistance to potential drugs is one of the main challenges in treatment of chronic and progressive diseases. However, radiosensitization offers a promising solution to the situation. It involves the sensitization of the tissues/cells to the radiation by physical, chemical, or pharmacological means (Gallez, 2015). It has been reported that the treatment of glioblastoma mouse model with a folate-targeted NP-mediated kringle 1 domain of hepatocyte growth factor gene can significantly induce antitumor effects and improve the sensitivity to ionizing radiation by promoting checkpoint kinase-1–induced cell cycle arrest and inhibiting the activation of tyrosine kinase receptors, ataxia telangiectasia mutated−checkpoint kinase-2 pathway, and Ki-67 expression (Figure 4; Zhang et al., 2018). Similarly, NPs made of lipid–poly(hypoxic radiosensitized polyprodrug), IO conjugated with epidermal growth factor receptor (EGFR), and RE have also been reported to enhance the sensitivity of radiotherapy in glioma cells by increasing the oxidative stress (Bouras et al., 2015; Hua et al., 2018; Lu et al., 2019). The cotreatment of PEGylated-Au NPs with radiation improve sensitivity, resulting in enhanced DNA damage (Joh et al., 2013). However, compared to Au NPs, Ag NPs have more sensitization effect mediated through the promotion of autophagy (Liu et al., 2016). In brief, NPs enhance the radiosensitivity of brain cells by promoting autophagy and hypoxia-induced oxidative stress, suggesting that this combination could be effective in treatment of brain diseases.

### The Application of NPs in the Treatment of Brain Diseases and Disorders

#### Brain Tumor

Brain tumor involves malignant and benign types of tumor that affect the brain. Because of the complexity of the brain, not only the metastatic but also the growth of benign tumor can have detrimental outcomes. According to the 2018 report, there were more than 298,000 new cases of brain tumor worldwide (Bray et al., 2018). The progression of the disease is associated with cognitive dysfunction (Cramer et al., 2019). The pathology of the disease is not well classified, and its treatment is still uncertain. However, several studies have reported the therapeutic advantage of NPs in delivering potential antitumor drugs (Nance et al., 2014; Feng et al., 2017; Chen et al., 2018). The delivery of small interfering RNA by targeting several genes including sodium–potassium (Na-K)–chloride cotransporter 1, yes-associated protein 1, roundabout homolog 1, EGFR, and survivin using polymeric NPs can significantly reduce the growth and migration of glioblastoma cells in a selective manner (Kozielski et al., 2019). It has been shown that modified polymeric NPs loaded with herpes simplex virus type 1 thymidine kinase combined with ganciclovir can markedly reduce the viability of glioma cells and increase the survival of tumor-bearing mice (Mangraviti et al., 2015). The above data confirm the use of NPs for gene and drug delivery to target brain tumors.

#### BIs

BIs consist of rare but deadly infections caused by microorganisms such as bacteria, viruses, fungi, and parasites that trigger inflammation in the brain or surrounding tissues (Sarrazin et al., 2012). Bacterial and viral infections are the most common. These infections are characterized by acute to chronic inflammations, oxidative stress, and subsequently neuronal impairment. A report shows that treatment with nerolidol-loaded NPs can efficiently improve memory defects and stabilize the levels of reactive oxygen species (ROS) and activities of Na-K ATPase and acetylcholine esterase previously altered by *Trypanosoma evansi* infection in mice (Baldissera et al., 2017). Also, the encapsulation of elvitegravir drug into PLGA NPs improves its inhibitory effects on human immunodeficiency virus 1 (HIV-1)–infected human monocyte-derived microglia-like cells and mouse model without affecting the integrity of the BBB (Gong et al., 2020). The clustered regularly interspaced short palindromic repeats (CRISPR)/CRISPR-associated protein 9/gRNA-loaded magnetoelectric NPs have also been reported to inhibit HIV-1 infection in microglia cells, indicating the potential of NPs in transporting gene therapy (Kaushik et al., 2019). Studies also show that NPs can increase the bioavailability of anti-HIV drugs such as darunavir, indinavir, and efavirenz, thereby improving their ability to cross BBB and targetability of viral infections in the brain (Desai and Thakkar, 2018; Karami et al., 2019; Martins et al., 2019). Besides, cobalt phosphate and hydroxide NPs have also shown potential inhibitory effects on parasite-induced toxicity of granulomatous amoebic encephalitis caused by *Acanthamoeba castellanii* belonging to the T4 genotype (Anwar et al., 2019a). The conjugation of the antidiabetic drugs glimepiride, repaglinide, and vildagliptin with Ag NPs can significantly inhibit the *A. castellanii*–mediated BI by preventing encystation and cytotoxicity (Anwar et al., 2019b). Further studies indicate that the treatment with Au and zinc oxide (ZnO) NPs can effectively improve oxidant/antioxidant status and neuronal impairment by regulating different genes altered following *Schistosoma mansoni* infection in mice (Dkhil et al., 2015; Bauomy, 2020). Similarly, Au NPs have been shown to reduce herpes simplex virus-1 infection–associated neurological defects by attenuating Aβ peptides aggregation and β-secretase activities (I et al., 2020). Otherwise, drug delivery with NPs has also shown enormous potential in treating cerebral tuberculosis, encephalitis virus infection, and amoebic meningoencephalitis infections in mouse models (Marcianes et al., 2017; Rajendran et al., 2017; LaBauve et al., 2018). Overall, the use of NPs in BI could reduce the cytotoxic effects and improve the efficiency of the treatments.

#### TBI

TBI is the leading cause of trauma-associated disabilities and deaths worldwide, estimated to affect more than 69 million people each year (Dewan et al., 2018). Accidents, sports, and gunshot are among the common causes of the TBI. The condition is associated with elevated levels of melanin in CSF that results in the promotion of oxidative stress and metabolic defects (Seifman et al., 2008). TBI patients have high levels of inflammatory markers in the CSF, which can be correlated with the severity of their condition (Kerr et al., 2018). TBI has been identified to be among the risk factors for neurodegenerative diseases including AD and Parkinson disease (PD) (Guo et al., 2000; Fleminger et al., 2003). Encapsulation of brain-delivered neutrophic factor, stromal cell–derived factor-1, cerebrolysin, and ROS-reactive agents into the NPs have been reported to be effective in improving neurological impairments resulting from TBI in mouse models (Ruozi et al., 2015; Khalin et al., 2016; Yoo et al., 2017; Zamproni et al., 2017). A recent study reports that treatment of TBI rat model with cerium oxide (CeO_2_) NPs can significantly reduce brain damage by restoring the cognitive abilities and promoting antioxidant properties (Bailey et al., 2020). It has been reported that the administration of immunomodulatory NPs can markedly improve motor impairments and reduce inflammatory and edema in TBI mouse model (Sharma et al., 2020). A risk analysis study reports that consumption of coffee in midlife reduces the risk of development of AD later in life, indicating that coffee has neuroprotective properties (Eskelinen et al., 2009). The NPs of coffee can be synthesized by boiling the coffee in water, filtrating to remove oil and large particles, followed by sonication (Ratliff et al., 2019). In TBI mouse model, treatment with nano-coffee can effectively improve behavioral characteristics and stabilize the levels of glycogen synthase kinase-3 (GSK-3) and poly(ADP-ribose) polymerase, the key biomarkers for apoptosis and cellular damage (Ratliff et al., 2019). In summary, NPs offer a crucial option for delivering drugs and reduce TBI-associated neurological damage by inhibiting apoptosis, inflammation, and oxidative stress.

#### Ischemic Stroke (IS)

Stroke is one of the primary causes of death and disability worldwide. IS is the most common type of stroke accounting for more than 79% of all stroke cases reported in 2017 (Virani et al., 2020). Almost 53% of new cases of IS reported in 2016 occurred in people between the ages of 44 and 70 years (Lindsay et al., 2019). The condition is characterized by the blockage of blood vessel as a result of blood clot or fat deposition. The progression of the disease is associated with several mechanisms including excitotoxicity, oxidative stress, and inflammation, which causes damage to cells and tissues (Castillo et al., 1999; Suwanwela et al., 2006; Kelly et al., 2008; Chehaibi et al., 2016). A recent study indicates that treatment of murine models of IS with selenium (Se) NPs can efficiently suppress neurodegenerative properties by regulating autophagy, inflammation, and oxidative stress through the upregulation of Unc-51–like autophagy activating kinase-1 and Wnt3a and suppression of Jack2/Stat3 and mTORC1 signaling cascades (Amani et al., 2019). Another study demonstrates that betulinic NPs improve the transportation of glyburide in BBB, resulting in enhanced antiedema and antioxidant properties in IS mice (Deng et al., 2019). Moreover, AMB3100-conjugated, size-shrinkable NPs have also been reported to facilitate the delivery efficiency of glyburide and reduce its toxicity (Guo et al., 2018). Besides, it has also been shown that the administration of polyhydroxylated fullerene NPs can significantly suppress brain damage by alleviating antioxidant status and nitric contents in IS mouse model (Vani et al., 2016). The protective effect of fullerene NPs is also linked with the downregulation of aquaporin-1 protein resulting in inhibition of edema (Darabi and Mohammadi, 2017). It has also been reported that treatment of IS rat model with melanin NPs can significantly inhibit the ROS and reactive nitrogen species–induced brain damage (Liu et al., 2017). So et al. (2019) showed that the administration of acetate-loaded liposomal NPs can markedly reduce microglial stimulation and chronic inflammation without affecting oxidative stress, apoptosis, and neurogenesis processes. Overall, the above information suggests that NPs can help in treatment of IS by acting as a delivery vehicle for drugs and by directly affecting the mechanism leading to the progression of the disease.

#### Amnesia

Amnesia is a medical condition characterized by memory loss, which results from either brain diseases or injury. Most common risk factors for the disease are substance abuse, toxicity, brain diseases, head injury, and blood loss (Langer, 2019). Currently, there are no specific drug treatments for the disease. Similar to other brain diseases, targeting amnesia is challenged by the drugs-associated protection mechanisms of the brain. A previous study reports a significant increase in memory recovery rate following the treatment with PS-80–coated rivastigmine CS NPs in mouse model of scopolamine (SC)–induced amnesia (Nagpal et al., 2013b). Similarly, nerve growth factor–loaded PBCA NPs modified with PS-80 reduce amnesic activities and improve cognitive functions in amnesia rat models (Basel et al., 2005). Besides, treatment with galantamine-loaded thiolated CS NPs can also restore memory defects in amnesia animal model (Sunena et al., 2019). In contrast, the gallic acid–loaded CS NPs coated with PS-80 and ZnO NPs show no significant improvement in memory loss induced by SC treatment in mice as compared to the administration of their corresponding pure drugs; however, these NPs can be used to enhance brain delivery of potential drugs (Nagpal et al., 2013a; Yadav et al., 2018). Collectively, NPs have shown improved drug-delivery efficiency; however, more studies are needed to investigate their effects in the treatment of amnesia.

#### ASD

ASD is a developmental disorder characterized by behavioral and communication difficulties. Individuals with ASD have poor communication skills as well as limited and repetitive behavioral patterns and interests (Bejerot and Nordin, 2014). The symptoms are usually seen in early childhood and progress to adulthood; however, with proper support and interventions, some difficulties can be camouflaged (American Psychiatric Association, 2013). The prevalence of the disorder in United States is 1/59 for 8 year-old children (Baio et al., 2018). In Norway, the prevalence of the ASD has been reported to increase, with more effect observed in preschool children ≤ 5 years old compared to school children aged 6–16 years (Özerk and Cardinal, 2020). Despite the lack of global data, there is a need for developing novel treatment options for the disorder. In valproic-induced ASD rats, treatment with nano-hesperetin could restore behavioral defects and inhibit inflammation and oxidative stress activities (Khalaj et al., 2018). Alternatively, prenatal exposure of mice with titanium dioxide (TiO_2_) NPs induces ASD-like behavioral impairment in offspring; however, the compound has no physiological effects (Notter et al., 2018). In brief, the above information indicates that NPs have potential in delivering drugs; however, more studies are needed to assess the side effects of the NPs.

#### Amyotrophic Lateral Sclerosis (ALS)

ALS is a group of progressive neurons-targeting degenerative diseases that affect the central nervous system (CNS), resulting in motor malfunctions and paralysis. The disease mostly affects children from 2 to 5 years, and in most cases, the death occurs within 5 years as a result of respiratory paralysis (Brown and Al-Chalabi, 2017). Currently, there are no cures for the disease. Compared to healthy individuals, ALS patients have a high rate of oxidative stress (Chico et al., 2018), neurotoxicity (Lam et al., 2016), and inflammation (Keizman et al., 2009). It has been reported that treatment with CeO_2_ NPs can effectively improve muscle activities and survival of ALS-induced mouse models by reducing oxidative stress–induced damage (DeCoteau et al., 2016). Similarly, treatment with Au NPs loaded with an inhibitor of hypoxia-inducible factor FM19G11 has been shown to promote the differentiation and proliferation of epidermal stem progenitor cells by elevating the associated genes in ALS mouse model (Marcuzzo et al., 2019). Moreover, treatment with adapalene-loaded poly(lactic acid)–poly(ethylene glycol) NPs induces neuroprotection and improves survival and motor functioning in ALS mice by stimulating retinoid signaling pathway (Medina et al., 2020). Together, these data imply that NPs have a great potential in improving the efficiency and transporting ASL drugs.

#### AD

AD is a common type of dementia characterized by aging-related progressive degeneration of neurons resulting in reduced cognitive ability and other neuropathological features. According to the 2016 data, at least one person develops the disease after each 66 seconds in America, and the number is expected to increase abruptly in the coming years (Alzheimer’s Association, 2016). The accumulation of Tau proteins is among the pathological features associated with the progression of neurodegenerative diseases including AD (Nam et al., 2020; Tagai et al., 2020). A recent study shows that protein-capped cadmium sulfide and IO NPs can effectively inhibit the polymerization and fibrillization of Tau proteins with the inhibition rates of 63 and 49%, respectively (Sonawane et al., 2019). Another pathological feature of AD is the accumulation of Aβ, which results into the reduced Aβ-binding capacity and formation of plaques (Hansson et al., 2009; Esparza et al., 2013). GSK-3, a serine/threonine kinase, has been shown to participate in the production of Aβ and hyperphosphorylation of Tau proteins and subsequently the progression of AD (Qu et al., 2014). Further evidences indicate that GSK-3 works with histamine deacetylase (HDAC) proteins to regulate neuronal activities (Chen et al., 2010; Bardai et al., 2012). Correspondingly, the inhibitors of GSK-3 and HDAC have been reported to be effective in suppressing AD (Green et al., 2008; De Simone et al., 2019; Soares Romeiro et al., 2019). The loading of nicotinamide, an HDAC inhibitor into the SL NPs, can significantly reduce cognitive impairment associated with AD by reducing the phosphorylation of Tau proteins in rat model (Vakilinezhad et al., 2018). Alternatively, the treatment of 5XFAD mice with vitamin D–binding protein–loaded PLGA NPs attenuates cognitive defects by inhibiting Aβ binding and accumulation (Jeon et al., 2019). Au NPs have also been reported to induce cytoprotective effects in AD rat model by promoting anti-inflammatory responses and improving antioxidant status (Dos Santos Tramontin et al., 2020). Furthermore, surface-coated Au NPs have also been shown to reduce Aβ aggregation, with the effect varying with the diameter and surface chemistry of the NPs (Moore et al., 2017). It has been reported that Au NPs with negative surface potential significantly reduce Aβ fibrillization and associated neurotoxicity in AD model (Liao et al., 2012). Furthermore, a recent study suggests that Au NPs with smaller size are more effective in suppressing Aβ fibrillization compared to the larger ones (Gao et al., 2017). Collectively, the data above signify that NPs can be used to deliver drugs targeting peptides dysregulated in AD such as Aβ more efficiently and effectively.

#### PD

PD is one of the most common types of neurodegenerative disorder with high prevalence in adults older than 50 years. Data show that more than 6.1 million people had PD in 2016, which is an increase of 2.4-fold from 1990 (GBD 2016 Parkinson’s Disease Collaborators, 2018). The disease is characterized by the loss of substantia nigra dopaminergic neurons and formation of Lewy bodies (LBs) and symptomized by motor and non-motor defects (Forno, 1996). Both genetics and environmental factors play a crucial role in the progression of the disease. The LBs contain α-synuclein aggregates (Spillantini et al., 1997, 1998), which contribute to the progression of the disease by facilitating neuronal loss and sensitivity to stresses (Cooper et al., 2018). α-Synuclein further participates in the promotion of apoptosis (Lee et al., 2001), inflammation (Chatterjee et al., 2020), and suppression of neuronal stem cell differentiation (Oliveira et al., 2015). A recent study indicates that α-synuclein is highly expressed in plasma and serum of PD patients compared to healthy individuals and suggests the possibility of using the protein as a diagnosis indicator for the disease (Chang et al., 2020). With respect to NPs, it has been reported that treatment with α-synuclein short-hairpin RNA-loaded magnetic IO NPs coated with oleic acid can efficiently improve motor dysfunction in PD mouse model by reversing α-synuclein–mediated elevation of apoptotic markers Bcl-2–associated X protein and p53 and suppression of B-cell lymphoma 2 (Niu et al., 2017). Study also shows that microRNA-124–loaded polymeric NPs are effective in repairing motor defects and alleviating PD symptoms (Saraiva et al., 2016). Alternatively, ceria NPs can also reduce ROS levels in PD mouse model (Kwon et al., 2018). Besides, treatment with iron (Fe) chelation NPs modified with zwitterionic poly(2-methacryloyloxyethyl phosphorylcholine) and HIV-1–transactivating transcriptor to delay its saturation in blood and increase its *in vivo* lifetime can reverse PD symptoms more effectively compared to individual treatments (Wang et al., 2017). Further study reveals that treatment of alkaline reserpine–induced PD mouse model with Au NPs can significantly reverse behavioral defects and improve antioxidant status and neuronal survival (da Silva Córneo et al., 2020). Moreover, the treatment of PD-induced mouse model with nanodopamine drugs also improves motor defects with low toxicity as compared to pure levodopa, a primary drug used for the treatment of PD (Vong et al., 2020). Likewise, metformin-loaded polydopamine NPs promote anti-inflammatory, antiapoptotic, and antioxidative properties associated with the proteolytic degradation of phosphorylated serine 129 of α-synuclein protein induced by targeting a histone-lysine N-methyltransferase enzyme known as the enhancer of zeste homolog 2 (Sardoiwala et al., 2020). Other NPs and nanodrugs that have been reported to have significant potential in the treatment of PD by regulating oxidative stress and inflammation including vitamin E–loaded naringenin nanoemulsions (Gaba et al., 2019), selegiline CS NPs (Sridhar et al., 2018), borneol and lactoferrin comodified NPs (Tang et al., 2019), resveratrol NPs (Palle and Neerati, 2018), and Cerium NPs (Hegazy et al., 2017). In summary, NPs and nanodrugs have great potential in treatment of PD because of their role in the regulation of inflammation, oxidative stress, apoptosis, α-synuclein activities, and the downstream effects in motor and non-motor dysfunctions.

#### Huntington Disease (HD)

HD is a progressive neurodegenerative disease of autosomal dominant origin characterized by motor, cognitive, and psychiatric impairments. Genetically, the disease occurs because of the mutation in huntingtin gene indicated by the extension of polyglutamate repeats in exon-1, the event that leads to the posttranslational-mediated functional defects of its downstream protein (Langbehn et al., 2004). High rate of tryptophan metabolism, inflammation, oxidative stress, excitotoxicity, and gene dysregulation has been established as key molecular processes associated with the progression of the disease in patients and animal model (Augood et al., 1997; Shin et al., 2005; Stoy et al., 2005; Sánchez-López et al., 2012; Hsiao et al., 2013). The analyses of brain autopsy from HD patients indicate a significant reduction of Se, an essential metal with protective properties against cytotoxicity and redox imbalance (Lu et al., 2014). Alternatively, a recent study reports that Se, iron, and chromium are among the essential elements that are considerably elevated in the blood samples of HD patients compared to normal individuals (Squadrone et al., 2020). In *Caenorhabditis elegans*, treatment with low doses of Se NPs reverses brain condition by improving oxidative status and inhibiting the aggregation of huntingtin proteins, suggesting the potential of the compound in the treatment of HD (Cong et al., 2019). Similarly, evidence shows that TiO_2_ NPs have the ability to catalyze the oxidation of methionine on the N-terminal domain of the mutant huntingtin protein, thereby forming a sulfoxide and preventing the aggregation of the protein (Ceccon et al., 2019). It has also been shown that the loading of thymoquinone into the SL NPs markedly suppresses the progression of HD by increasing the activity of ATPase enzymes and reducing the production of inflammatory markers and the nuclear translocation of phosphorylated nuclear factor κB in rat model (Ramachandran and Thangarajan, 2018). Moreover, the encapsulation of peptide-based polyglutamate aggregation inhibitors into PLGA NPs can enhance their protective effects in Neuro 2A and PC12 cellular models as well as its biocompatibility in *Drosophila* model of HD (Joshi et al., 2019). In both neuronal cell and mouse model, poly(trehalose) NPs have also been reported to be extremely efficient in inhibiting the progression HD by suppressing the accumulation of mutant huntingtin protein (Debnath et al., 2017). The alteration of cholesterol metabolism has also been reported in the animal model of the disease (Marullo et al., 2012). Specifically, HD is linked with the alteration in the levels of 24S-hydroxycholesterol, a vital cholesterol metabolite produced by the hydroxylation reaction catalyzed by cholesterol-24 hydrolase (Leoni et al., 2013). A recent study shows that the elevation of the enzyme is crucial for the treatment of the disease as it facilitates the proteasomal and autophagy-mediated clearance of mutant huntingtin aggregates (Kacher et al., 2019). Besides, evidence shows that treatment with cholesterol-loaded glycopeptide-modified polymeric NPs can reverse behavioral and cognitive defects in HD mice (Valenza et al., 2015). In analyzing the nose to brain delivery, Passoni et al. (2020) reveal that liposomal NPs are effective in delivering cholesterol via this route in HD mouse model, confirming its potential in the treatment of HD. In brief, these above evidences confirm the neuroprotective role of NPs and their potential in the treatment of HD by targeting key mechanisms involved in the progression of the disease.

#### MS

MS is a neurological disorder of the CNS and a common cause of disability in young adults. The disease affects more than 2.2 million people worldwide, and its prevalence has increased significantly in many regions (GBD 2016 Multiple Sclerosis Collaborators, 2019). Currently, there are no effective cures for the disease; however, several drugs are used to treat/reduce the symptoms of the disease especially in initial stages. The main features of the disease reported to occur in early patients include cortical demyelination and meningeal inflammation (Bø et al., 2003; Lucchinetti et al., 2011). Recent studies demonstrate that SL NPs and CS NPs can potentially increase the bioavailability and neuroprotective effects of a relapsing-MS drug dimethyl fumarate in rat model (Ojha and Kumar, 2018; Ojha et al., 2019). Another study also suggests that glucocorticoids and inorganic–organic hybrid NPs can also be used to treat MS (Montes-Cobos et al., 2017). Moreover, the encapsulation of chondroitinase ABC 1 into porous silicon NPs counteracts the neuronal damage by facilitating remyelination in MS mouse model (Rezaei et al., 2020). Together, these data suggest that NPs can be used to improve the efficiency and bioavailability of potential MS drugs.

#### Epilepsy

According to the International League Against Epilepsy, epilepsy is regarded as a brain disease characterized by (i) two unprovoked or reflex seizures occurring over 24 h apart; (ii) one unprovoked/reflex seizure and at least 60% probability of further seizures to occur over the next 10 years, after two unprovoked seizures; and (iii) the diagnosis of epilepsy syndromes (Fisher et al., 2014). The disease can occur at all ages; however, it is common in children and adults. The activation of astrocytes and microglia plays a key role in the progression of the disease (Shapiro et al., 2008; Najjar et al., 2011; Bedner et al., 2015). The treatment of epilepsy has been hindered by the low bioavailability and the delivery of the drugs to the brain. Curcumin has proven to be potential in the treatment of epilepsy because of its ability to suppress cognitive deficit and glial activation and promote antioxidant and anti-inflammatory properties (Kaur et al., 2015). In a mouse model of chronic epilepsy, the incorporation of curcumin with CS-alginate STPP NPs significantly increases its corresponding effects on cell death, cognitive defects, and glial activation, consistently with the solubility of the compound (Hashemian et al., 2017). Curcumin-loaded SL NPs induce neuroprotective effect by reducing apoptosis via upregulation of erythropoietin and klotho, reduction of tumor necrosis factor-α (TNF-α), and the subsequent activation of P38 MAPK pathways (Mansoor et al., 2018; Huang et al., 2020). It has also been shown that treatment with piperine-loaded CS-STPP NPs strongly inhibits the progression of epileptic symptoms by suppressing cell death and astrocyte stimulation compared to non-loaded piperine-treated mice (Anissian et al., 2018). A previous study also reports that treatment of epileptic rat model with pluronic P85-coated PBCA NPs alleviates the effects of P-gp in phenyltoin resistance and increases its bioavailability (Fang et al., 2016). The loading of carbamazepine (an anticonvulsant drug used to treat epilepsy) into the poloxamer 188–coated PLGA NPs also improves the drug effect in isoniazid-induced epilepsy rat model compared to the administration of free drug (Zybina et al., 2018). Further evidence suggests that the treatment with quercetin-conjugated IO-β-cyclodextrin NPs can markedly enhance the therapeutic effect of quercetin in epileptic mouse model (Hashemian et al., 2019). Together, the data above suggest that the incorporation of potential epilepsy drugs into NPs improve their sensitivity and efficiency.

## Approved Nanodrugs and Ongoing Clinical Trials

The success of NPs in clinical trials is evidenced by more than 250 US Food and Drug Administration–approved nanodrugs available on the market. Some of the interesting drugs include Doxil (doxorubicin HCL liposome injection), Invega Sustenna (paliperidone palmitate), DepoCyt (liposomal cytarabine), and Plegridy (PEGylated interferon β-1a) used for the treatment of multiple myeloma, schizophrenia, lymphomatous meningitis, and MS, respectively (Ventola, 2017). Liposomal formulation is the most common nanodrug available in the market so far, implicating more than 33% of drugs (D’Mello et al., 2017). In addition, numerous clinical trials have been conducted to identify the applicability of NMs in clinical settings. Previous study reports that glioblastoma patients treated with magnetic NPs and reduced radiotherapy have improved overall survival compared to conventional therapy–treated counterparts (Maier-Hauff et al., 2011). Besides, NPs also help to reduce toxicity caused by conventional drug (Eckes et al., 2011). It is worth noting that magnetic NPs also show 70% chemotherapy (temozolomide)–delivering capability and distribution in intracranial tumor region in pet dogs (Young et al., 2018). Moreover, recent studies reveal that treatment with omega-3 fatty acids and curcumin NPs significantly reduces inflammation in migraine patients by suppressing the expressions of TNF-α, intercellular adhesive molecule 1, and cooxygenase-2/inducible nitric oxide synthase (Abdolahi et al., 2017, 2019; Soveyd et al., 2018; Table 1). Together, the above evidences indicate that NPs have enormous potential in brain diseases by facilitating drug delivery, inducing synergistic effects, and reducing drug toxicity.

**TABLE 1:** Clinical trials for NPs-based treatments for brain diseases and disorders.

Type of NPs	Medical conditions	Effects	Mechanisms	References
Magnetic IO NPs + reduced radiotherapy	Glioblastoma multiforme	Improves patients’ overall survival	Suppression of tumor growth by increasing Caspase-3, heat shock protein, and programmed death ligand 1 levels	Maier-Hauff et al., 2011; Grauer et al., 2019
Nano-curcumin + ω-3 fatty acids	Migraine	Reduces recurrent headaches	Suppression of gene expressions of pro-inflammatory TNF-α, intercellular adhesion molecule 1, and cyclooxygenase-2/inducible nitric acid	Abdolahi et al., 2017, 2019; Soveyd et al., 2018
Ag NPs	Acute occlusive hydrocephalus	Improves patients’ status	Prevents catheter-associated ventriculitis	Lackner et al., 2008
Ultrasmall magnetic IO	IS	Enhances inflammatory cytokines targetability	Promotes macrophage infiltration	Saleh et al., 2007

**NPs, nanoparticles; Ag, silver; IO, iron oxide; IS, ischemic stroke.**

## Conclusion and Future Direction

The incorporation of nanotechnology in medical field helps to improve the diagnosis and treatment of different diseases by increasing the sensitivity of equipment and different parameters of the drugs, thereby enhancing their efficacy. Because of the ability of NPs to cross the BBB, these compounds provide a potential option for diagnosing and treating the brain diseases and disorders, which have proven to be challenging for many years. However, to ensure the effectiveness and efficiency of these particles, further studies are needed to determine their toxicity and bioaccumulation in clinical settings. Some of these NPs cause deterioration of the brain functioning and increase oxidative stress (Table 2). Therefore, the use the NPs with therapeutic usefulness and low toxicity should be prioritized to achieve high outcome and prevent further damage to the brain. In addition, it is important to enhance their sensitivity to target specific biomarkers by improving the formulation with specific antibodies. After further exploration, the potential of nanotechnology in the treatment of brain diseases and disorders will be limitless.

**TABLE 2:** Possible side effects associated with metal NPs.

Type of NPs	Animal models	Effects	References
Carbon black	Female pregnant mice	Impairs neurons development on infants and causes irreversible brain damage on mother	Zhang et al., 2019
ZnO	Male Wistar rats	Impairs neuronal mitochondria functions by ROS production	Liu et al., 2020a
	Swiss albino mice	Induces motor defects	Yaqub et al., 2020
Aluminum oxide	Male Wistar rats	Increases inflammatory responses and oxidative stress	Liu et al., 2020b
IO	Mice	Impairs motor and behavioral functions	Manickam et al., 2019
TiO_2_	Mice	Damages dopaminergic neurons	Heidari et al., 2019
CuO	Mice	Reduces motor, behavioral, and locomotor activities	Ouni et al., 2020
Ag	Rats	Induces myelin sheath damage due to ROS	Da̧browska-Bouta et al., 2019

**ZnO, zinc oxide; IO, iron oxide; TiO_2_, titanium oxide; CuO, copper oxide; Ag, silver; ROS, reactive oxygen species.**

## Author Contributions

EN, Y-ZW, LQ, D-DW, S-FD, and X-YJ: conceptualization. EN, Y-ZW, and LQ: data curation. X-YJ and D-DW: funding acquisition. EN, Y-ZW, LQ, YH, BA, TL, MZ, and E-SJ: writing–original draft. S-FD, J-SW, D-DW, and X-YJ: visualization and supervision. EN, Y-ZW, LQ, and D-DW: editing. All authors contributed to the article and approved the submitted version.

## Conflict of Interest

The authors declare that the research was conducted in the absence of any commercial or financial relationships that could be construed as a potential conflict of interest.

## Abbreviations

BBB: blood–brain barrier
NPs: nanoparticles
P-gp: p-glycoproteins
SL: solid lipid
CS: chitosan
PEG: polyethylene glycol
IO: iron oxide
Ag: silver
STPP: sodium tripolyphosphate
Au: gold
A β: amyloid β
MI: molecular imaging
PS-80: polysorbate 80
RE: rare-earth
UCH-L1: ubiquitin-C-terminal hydrolase-L
EGFR: epidermal growth factor receptor
CNS: central nervous system
ROS: reactive oxygen species
Se: selenium
CuO: copper oxide
ZnO: zinc oxide
CeO_2_: cerium oxide
BI: brain infections
TBI: traumatic brain injury
CSF: cerebral spinal fluid
IS: ischemic stroke
SC: scopolamine
ASD: autism spectrum disorder
TiO_2_: titanium dioxide
ALS: amyotrophic lateral sclerosis
AD: Alzheimer disease
GSK-3: glycogen synthase kinase-3
HDAC: histamine deacetylase
PD: Parkinson disease
LB: Lewy bodies
Na-K: sodium–potassium
PBCA: poly(butyl cyanoacrylate).
